# Additional Treatment With Heparin and Warfarin Also Improves T2DM and Partially Resolves Ascending Aortic Thrombi: A Case Report

**DOI:** 10.7759/cureus.95390

**Published:** 2025-10-25

**Authors:** Hidekazu Takeuchi

**Affiliations:** 1 Internal Medicine (Cardiology), Takeuchi Naika Clinic, Ogachi-Gun, JPN

**Keywords:** ascending aortic thrombus, heparin-warfarin treatment, homocysteine, pulmonary vein thrombosis, stroke, t2dm

## Abstract

Type 2 diabetes mellitus (T2DM) is a common disease and a clinically significant problem. When patients are treated with antidiabetic medications, issues related to target blood glucose levels can arise. Although achieving lower glucose targets is beneficial, it increases the risk of hypoglycemia. Because conventional treatments are often ineffective in addressing this issue, new strategies are needed. Heparin-warfarin treatment partially resolved portal vein thromboses (PVTs); subsequently, I unexpectedly observed that heparin-warfarin therapy could ameliorate mild to moderate T2DM, reducing the need for antidiabetic medications to one or none. One to two weeks after initiating heparin-warfarin treatment, I had to reduce the use of diabetes medications to one or zero to continue the therapy and avoid hypoglycemia. It remains unclear whether additional heparin-warfarin treatment can further improve glycemic control in patients who require only one diabetes medication. A 72-year-old male with a history of cerebral infarction and T2DM was initially treated with heparin-warfarin and later received additional heparin-warfarin therapy to resolve PVTs. The patient underwent transesophageal echocardiography and cardiac CT to assess chronic thrombi. Heparin-warfarin treatment improved T2DM and partially resolved PVTs and thrombi in the ascending aorta (AAo). With additional therapy, T2DM further improved, allowing a reduction in diabetes medications from 3 to 0. After discontinuation of diabetes medication, the patient experienced neither hypoglycemia nor hyperglycemia, and blood glucose levels ranged from 90 to 120 mg/dL before meals. Some thrombi were partially resolved. Overall, additional heparin-warfarin therapy improved the patient’s T2DM, reduced the need for diabetes medications from three to none, and partially resolved PVTs and AAo thrombi. Heparin-warfarin therapy may represent a safer and more effective option for patients with T2DM.

## Introduction

The number of patients with type 2 diabetes mellitus (T2DM) worldwide is 0.5 billion, and this number is expected to increase to more than 2 billion in the coming decades [1], with further increases anticipated [2]. Patients with T2DM need to accept a lower quality of life, such as work restrictions, diet limitations, and insulin injections. Moreover, they have an increased risk of other atherosclerotic diseases, including acute myocardial infarction (AMI) and acute ischemic stroke (AIS). Additionally, T2DM can cause many complications, including central nervous system diseases such as Alzheimer’s disease [3] as well as retinopathy, diabetic kidney disease, and peripheral neuropathy as microvascular diseases. Although a glycemic goal of 80-110 mg/dL with insulin reduced mortality by 40% compared with a standard glycemic goal of 180-215 mg/dL, hypoglycemia occurred at 10- to 15-fold higher rates [4]. Methods to decrease blood glucose levels without a risk of hypoglycemia are needed.

Studies of thrombi retrieved from patients with AIS have revealed calcifications and collagen [5], indicating that these thrombi are chronic or old and that patients with AIS or AMI have chronic thrombi prior to the onset of AIS or AMI. Neurosurgeons often use the term “cardiogenic thrombi.” Although their observation is correct, they do not know what the thrombi are.

When a respiratory infection occurs, neutrophils produce neutrophil extracellular traps (NETs) to kill pathogens [6]. NETs form and stabilize thrombi in the fine pulmonary veins, preventing antigens from spreading throughout the body [7]. Retrieved thrombi and NETs are well studied; however, their relationships are unclear. Fine thrombi in the fine pulmonary vein might become increasingly large and produce pulmonary vein thrombi (PVTs), which can be detected by cardiac computed tomography (CT); expand into the left atrium (LA), which could be detected not by cardiac CT but by transesophageal echocardiography (TEE); and reach the wall of the LA [8]. Those large thrombi could be broken and released, potentially causing AIS and AMI via so-called retrieved thrombi. I reported that PVTs are common diseases in elderly patients with age-related diseases such as hypertension, dyslipidemia, T2DM, heart failure, angina pectoris, and atrial fibrillation [9] and that warfarin and direct oral anticoagulants (DOACs) partially resolved the PVTs and LA thrombi extending from the PVTs [9]; therefore, I used heparin-warfarin to resolve those thrombi completely.

I reported that 1 month of heparin-warfarin treatment improved mild-to-moderate T2DM, reducing the need for DM medications, sometimes to only 1 or 0 [10]. NETs promote inflammation by activating inflammatory cells, which can induce many diseases, including T2DM. NETs also damage endothelial function, promote the formation of microclots, obstruct blood flow, and induce a shortage of oxygen and nutrients; moreover, blood, including insulin, cannot reach end cells. Such conditions provoke oxidative stress. Heparin can alleviate NET-associated diseases by degrading NETs through histone collapse; thus, it can alleviate T2DM.

Additionally, inflammation and oxidative stress have recently been reported to be associated with a high occurrence of cardiovascular diseases (CVDs) in T2DM patients. Heparin might ameliorate T2DM and prevent CVD by reducing inflammation and oxidative stress, thereby improving microcirculatory function. However, it is unclear whether additional heparin-warfarin treatment can decrease the use of one DM medication to zero.

## Case presentation

The patient was a 73-year-old male who had T2DM and dyslipidemia, and an old cerebral infarction was examined to assess the presence of chronic thrombi, such as left atrial appendage thrombi and PVTs, using enhanced CT and TEE. His breath sounds were normal, and there were no crackles or wheezes. Heart sounds were clear, and the rhythm was regular without audible murmurs or friction sounds. He had paresis of his right leg, used a cane when he walked, and was talkative. His blood pressure was 124/83, his pulse was 67 and regular, and his respirations were 14. His brain natriuretic protein (BNP) concentration was 10.1 pg/mL (normal: <18.4 pg/mL). He had been treated with clopidogrel (75 mg: once a day) and cilostazol (100 mg: twice daily). He had also been treated with vildagliptin (50 mg twice daily), metformin (500 mg twice daily), and dapagliflozin (5 mg twice daily) for T2DM. The patient’s DM was well controlled with these three medications, and his glycated hemoglobin (HbA1c) level was 6.6% (normal: 4.6-6.2%).

Electrocardiography revealed a normal sinus rhythm, a normal axis, and slight depression of the ST. The serum D-dimer concentration was 1.0 μg/ml (normal: < 1.0 μg/ml), the activity of protein S was 101% (normal: 74-132%), and the activity of protein C was 106% (normal: 64-135%). The homocysteine concentration was 20.3 nmol/mL (normal: 5:15 nmol/mL). I checked the patient’s blood glucose levels three times a day (30 minutes before breakfast, lunch, and dinner).

**Figure 1:**
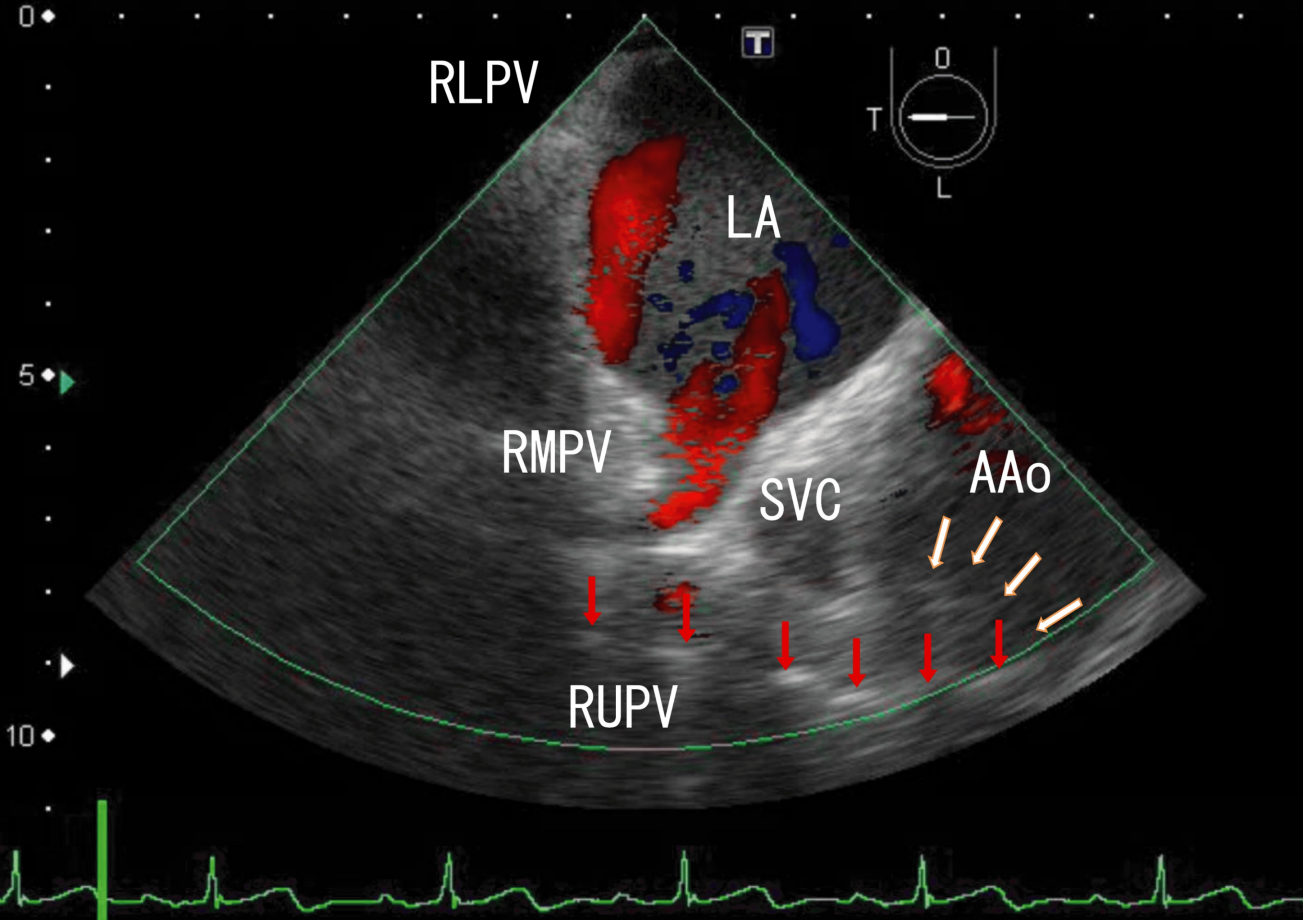
TEE images showing AAo thrombi and approaching several white parts TEE revealed large thrombi in the AAo with white areas, which appeared to connect to a series of white areas directed toward the RUPV (red arrows). There were AAo thrombi around the white thrombi (white arrows). Red areas in the AAo and LA indicate the blood flow in the AAo and from the RUPV and the RMPV, respectively. Thrombi inhibited red blood flow from the RMPV to the RLPV. TEE: transesophageal echocardiography, AAo: ascending aorta, LA: left atrium, SVC: superior vena cava, RLPV: right lower pulmonary vein, RUPV: right upper pulmonary vein, RMPV: right middle pulmonary vein

TEE revealed right lower pulmonary vein (RLPV) thrombi and white thrombi around the ostia of the right upper pulmonary vein (RUPV), which seemed to be connected to line-like white thrombi situated on the side of the superior vena cava (SVC) and reached white areas in the ascending aorta (AAo) thrombi (Figure 1). There were rather vague, large, whitish thrombi around the central white areas in the AAo thrombi (Figure 1; start). Video images revealed that the AAo thrombi moved with heartbeats; however, the RUPV thrombi and line-like thrombi did not (Video 1; start). Cardiac CT did not reveal AAo thrombi, whose characteristics are similar to those of LA thrombi that extend from PVTs.

**Video 1:** TEE video images showing AAo thrombi and approaching several white parts TEE video revealed large thrombi in the AAo with white parts, and the white parts seemed to connect to a series of white parts, which were directed to the RUPV. White parts in the AAo moved with heartbeats; however, areas near the white parts were not moved with heartbeats. They did not connect. Red areas in the AAo and LA indicate blood flow in the AAo and from the RUPV and the RMPV. Red areas in the AAo were very small, indicating the presence of large thrombi. Red blood flow from the RMPV was inhibited by thrombi from the RLPV, whose thrombi moved with heartbeats. The upper and lower areas of the AAo wall could not be clearly identified. The approximate thrombus size of the whitish AAo thrombi was 4 cm × 2 cm, and that of the dark LA thrombi was 5 cm × 4 cm. TEE: transesophageal echocardiography, AAo: ascending aorta, RUPV: right upper pulmonary vein, LA: left atrium, RMPV: right middle pulmonary vein, RLPV: right lower pulmonary vein

**Figure 2:**
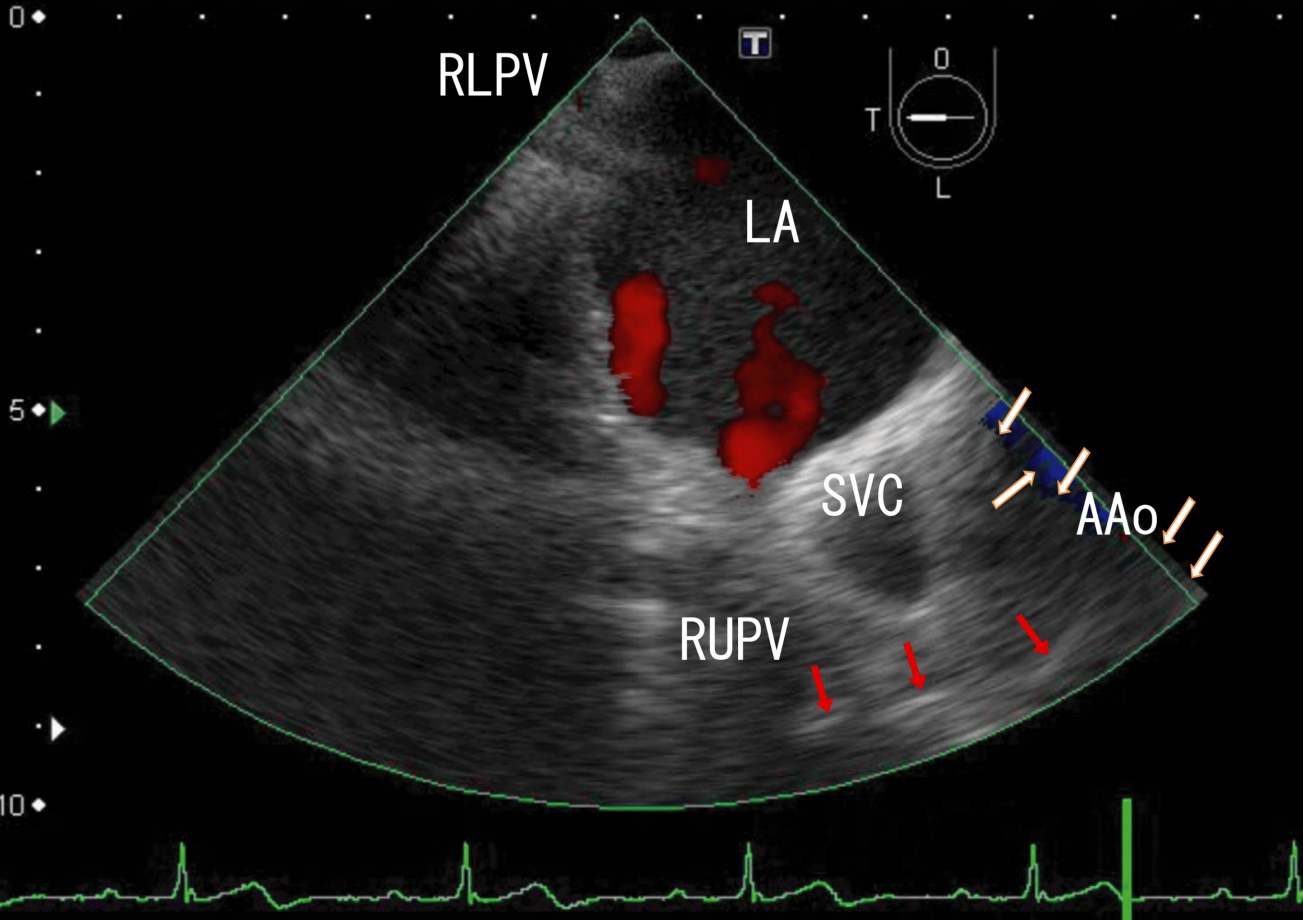
TEE images showing AAo thrombi and approaching several white parts TEE revealed large thrombi in the AAo with white parts (white arrows), and the white parts seemed to connect to a series of white parts, which were directed to the RUPV (red arrows). Red areas in the AAo and LA indicate blood flow in the AAo and from the RUPV and the RMPV. Thrombi inhibited red blood flow from the RMPV to the RLPV. The small red area represents blood flow from the RLPV. The LA thrombi from the RLPV became larger. TEE: transesophageal echocardiography, AAo: ascending aorta, LA: left atrium, SVC: superior vena cava, RLPV: right lower pulmonary vein, RUPV: right upper pulmonary vein, RMPV: right middle pulmonary vein

I treated the patient with a standard dose of heparin for the first three weeks, after which clopidogrel and cilostazol were continued. AAo thrombi were not affected, and the LA thrombi became slightly smaller (Figure 2 and Video 2; three weeks had passed from the start). However, two weeks after the start of this treatment, the patient’s blood glucose level decreased, and I needed to stop taking the two DM medications, vildagliptin and metformin. Heparin alone could not resolve thrombi; however, it could improve T2DM. I needed to decrease the use of two DM medications to avoid hypoglycemia and maintain heparin therapy. The serum D-dimer concentration was 1.0 μg/ml (normal; < 1.0 μg/ml), and his HbA1c was 6.8%.

**Video 2:** TEE video images showing AAo thrombi and approaching several white parts TEE video revealed large thrombi in the AAo with white areas, and the white areas appeared to connect to a series of white areas directed toward the RUPV. White parts in the AAo moved with heartbeats; however, areas near them did not. They did not connect. Red areas in the AAo and LA indicate blood flow in the AAo and from the RUPV and the RMPV. Red areas in the AAo were very small, indicating the presence of large thrombi. The two thrombi slightly inhibited red blood flow from the RMPV to the RLPV, whose thrombi moved with heartbeats. Red blood flow from the RUPV increased, indicating that LA thrombi decreased. The upper and lower areas of the AAo wall could not be clearly identified. The approximate thrombus size of the whitish AAo thrombi was 3 cm × 1.5 cm, and that of the dark LA thrombi was 5 cm × 3.5 cm. TEE: transesophageal echocardiography, AAo: ascending aorta, RUPV: right upper pulmonary vein, LA: left atrium, RMPV: right middle pulmonary vein, RLPV: right lower pulmonary vein

**Figure 3:**
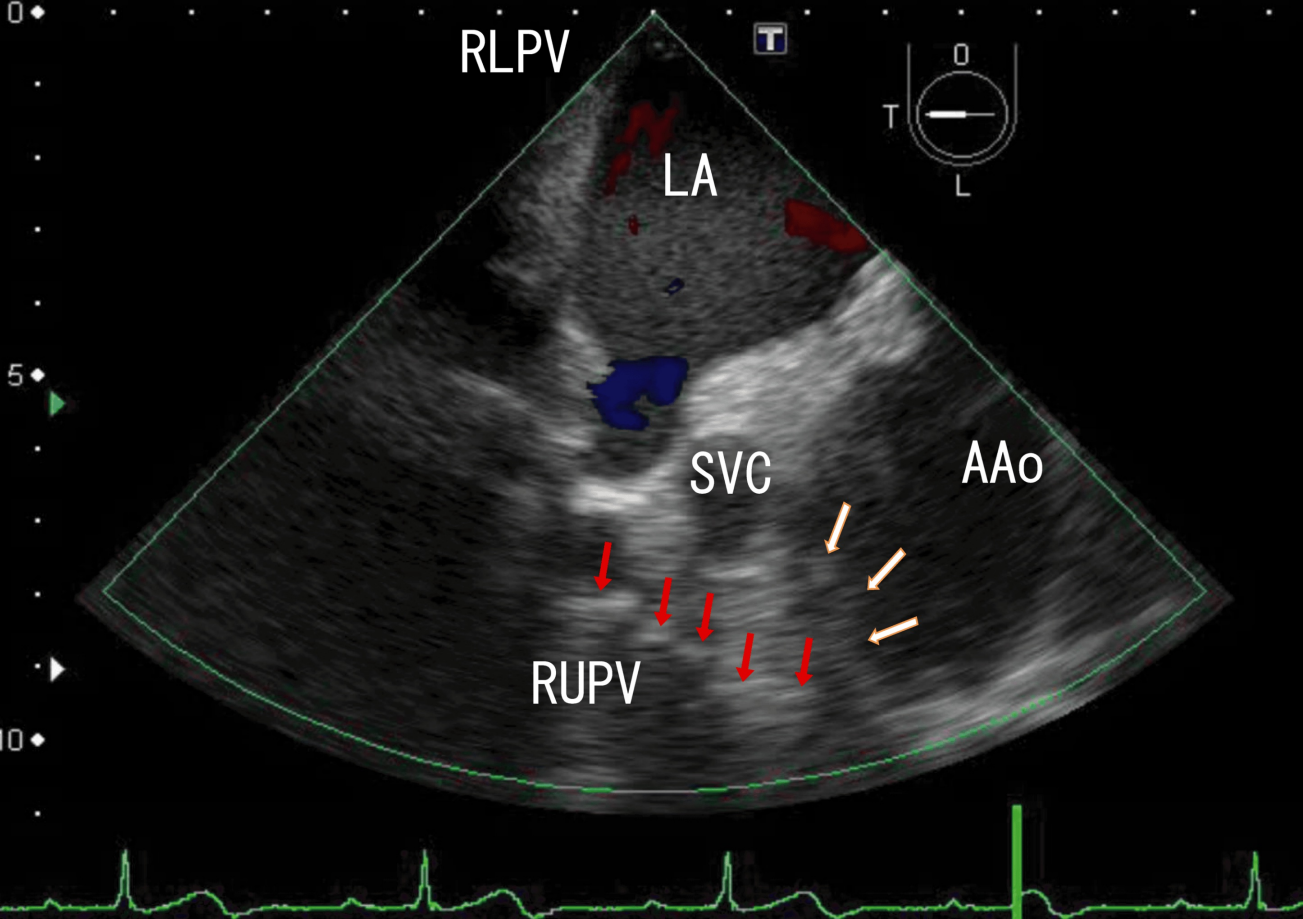
TEE images showing decreased AAo thrombi and approaching several white parts TEE revealed large thrombi in the AAo (white arrows), and a series of white parts remained, which were directed to the RUPV (red arrows). The blood flow in the AAo increased, indicating that the AAo thrombi were partially resolved. Blood flow from the RUPV and the RMPV did not change, indicating that the LA thrombi did not resolve, as shown in dark white. The LA thrombi from the RLPV disappeared. TEE: transesophageal echocardiography, AAo: ascending aorta, LA: left atrium, SVC: superior vena cava, RLPV: right lower pulmonary vein, RUPV: right upper pulmonary vein, RMPV: right middle pulmonary vein

I treated the patient for the next three weeks with standard doses of heparin-warfarin. AAo thrombi partially resolved (Figure 3 and Video 3; six weeks had passed from the start). After two weeks, the patient developed hypoglycemia; therefore, I needed to decrease the use of DM medication to zero. The last DM medication was stopped to avoid hypoglycemia and maintain heparin-warfarin therapy; therefore, the patient received no DM medications. He wanted to continue the treatment; however, he needed to be discharged for ten days for his social activities. During these ten days, he received warfarin alone.

**Video 3:** TEE video images showing AAo thrombi and resolved LA thrombi TEE revealed that the thrombi in the AAo group decreased in size, and the white areas appeared dark and small. The white parts in the AAo moved with heartbeats. Red and blue areas in the AAo were enlarged, indicating that the AAo thrombi resolved. Red blood flow from the RMPV and RUPV did not change, and the thrombi did not resolve. Red blood flow from the RUPV increased, indicating that LA thrombi decreased. The lower areas of the AAo wall could not be clearly identified. The approximate thrombus size of the whitish AAo thrombi was 1.5 cm × 1 cm, and that of the dark LA thrombi was 4.5 cm × 3.5 cm. TEE: transesophageal echocardiography, AAo: ascending aorta, RUPV: right upper pulmonary vein, LA: left atrium, RMPV: right middle pulmonary vein

**Figure 4:**
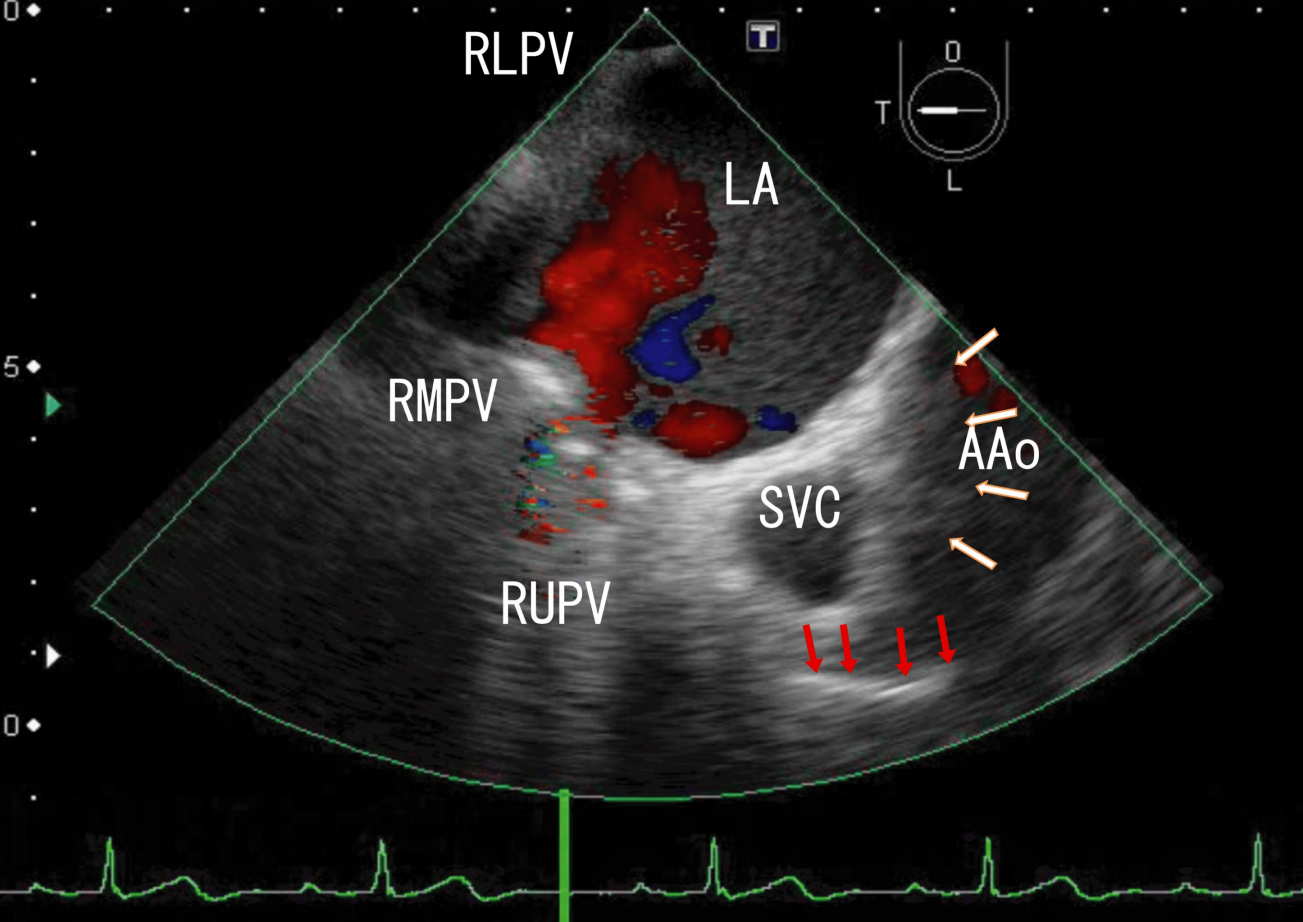
TEE images showing AAo thrombi and approaching several white parts TEE revealed small thrombi in the AAo (white arrows), with the attachment areas located on the upper side. Most thrombi in the AAo resolved, and the LA thrombi could have been small. The red blood flow from the RMPV increased slightly. The small red area represents blood flow from the RLPV. The LA thrombi from the RLPV became larger. Short, line-like white thrombi could be identified (red arrows). TEE: transesophageal echocardiography, AAo: ascending aorta, LA: left atrium, SVC: superior vena cava, RLPV: right lower pulmonary vein, RUPV: right upper pulmonary vein, RMPV: right middle pulmonary vein

I treated the patient for the next four weeks with standard doses of heparin-warfarin. AAo thrombi resolved (Figure 4 and Video 4; 2.5 months had passed from the start), and the effects on T2DM were maintained. The serum D-dimer concentration was 1.0 μg/ml, and his HbA1c was 7.2%.

**Video 4:** TEE video images showing AAo thrombi and approaching several white parts TEE video revealed small thrombi in the AAo and the series of white parts, which were directed to the RUPV. Red areas in the AAo and LA indicate the blood flow in the AAo and from the RUPV and the RMPV. The red and blue areas in the AAo were large, indicating the presence of large thrombi. The two thrombi slightly inhibited red blood flow from the RMPV to the RLPV, which moved with heartbeats. Red blood flow from the RUPV increased, indicating that LA thrombi decreased. The upper and lower areas of the AAo wall could not be clearly identified. The approximate thrombus size of the whitish AAo thrombi was 1 cm × 0.5 cm, and that of the dark LA thrombi was 5 cm × 3 cm. TEE: transesophageal echocardiography, AAo: ascending aorta, RUPV: right upper pulmonary vein, LA: left atrium, RMPV: right middle pulmonary vein, RLPV: right lower pulmonary vein

**Figure 5:**
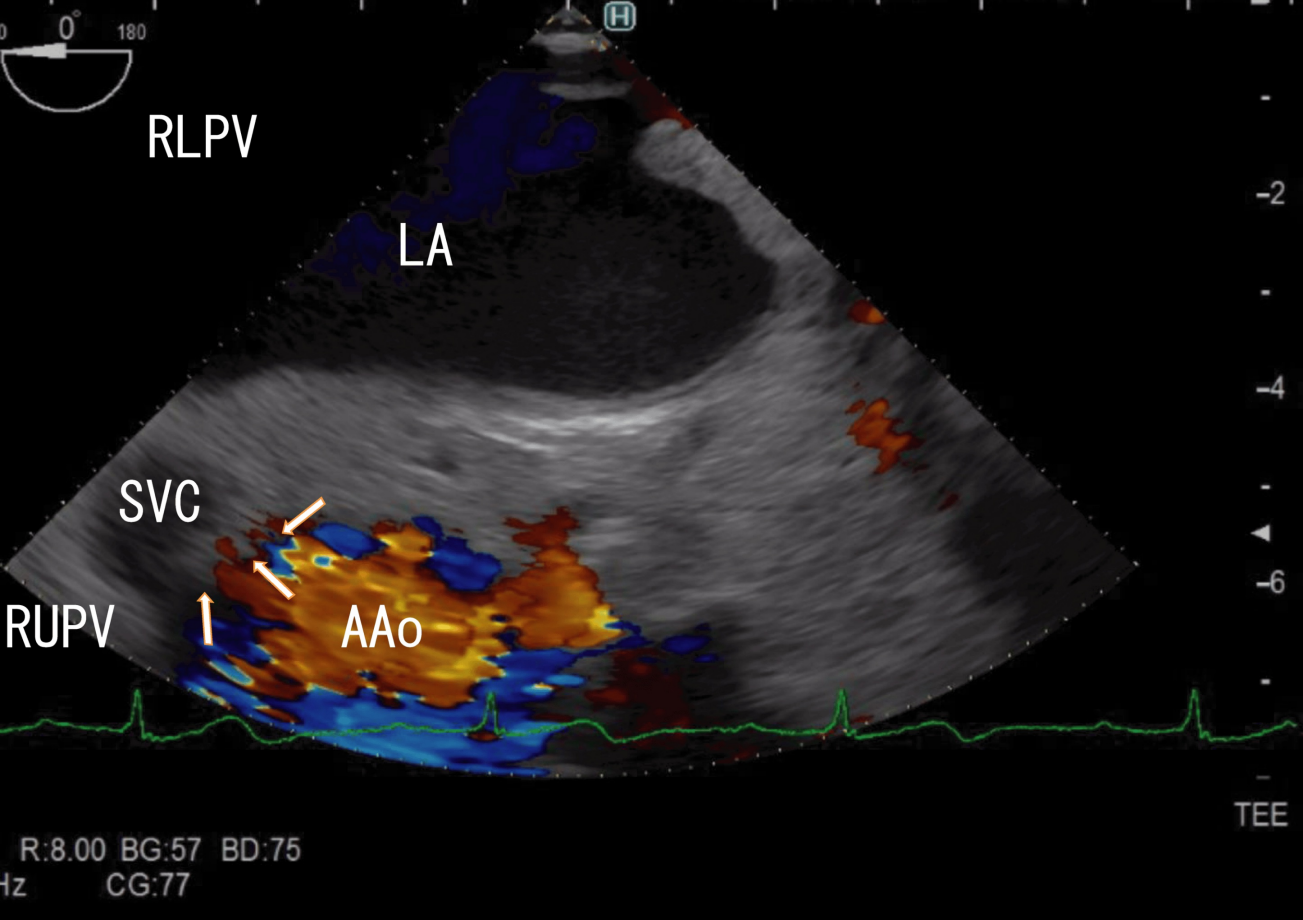
TEE images showing resolving AAo thrombi TEE revealed half of the AAo thrombi in red, blue, and yellow, indicating their resolution. On the upper right side of the AAo, there were a few AAo thrombi (white arrows). TEE: transesophageal echocardiography, AAo: ascending aorta, LA: left atrium, SVC: superior vena cava, RLPV: right lower pulmonary vein, RUPV: right upper pulmonary vein

For the next five months, he was treated with only a standard dose of warfarin. The TEE angle was different from the previous one. The TEE showed dark AAo thrombi (Figure 5 and Video 5; 7.5 months had passed from the start). His HbA1c was 7.0%.

**Video 5:** TEE video images showing resolving AAo thrombi TEE video revealed half of the AAo with blood flow, red, blue, and yellow, indicating the resolution of the AAo thrombi. On the upper right side of the AAo, there was a moving thrombus (15 mm × 10 mm). An approximate thrombus size of the old, whitish AAo thrombi was not observed, and there were no dark LA thrombi. TEE: transesophageal echocardiography, AAo: ascending aorta, LA: left atrium

**Figure 6:**
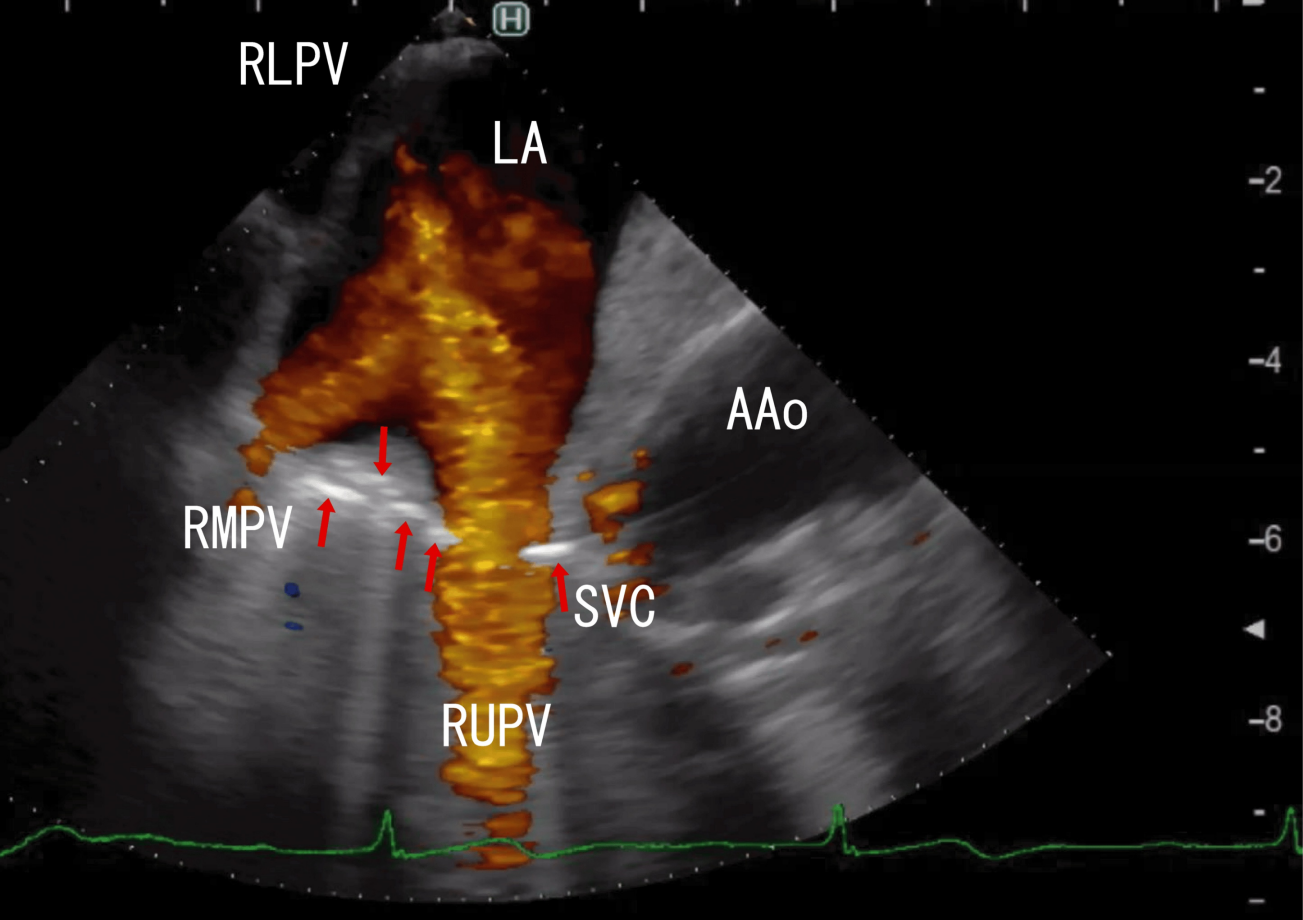
TEE images showing AAo thrombi and approaching several white parts TEE revealed no thrombi in the AAo. There were white thrombi around the ostia of the RMPV, which seemed to approach the wall of the AAo via line-like white thrombi (red arrows). The red areas in the SVC, located on the right side of the AAo, indicate blood flow in the SVC. The red areas in the LA appeared to have no obstruction. Red blood flow from the RMPV and RUPV moves straight up, indicating the absence of thrombi in the LA. TEE: transesophageal echocardiography, AAo: ascending aorta, LA: left atrium, SVC: superior vena cava, RLPV: right lower pulmonary vein, RUPV: right upper pulmonary vein, RMPV: right middle pulmonary vein

For the next four weeks, the patient was treated with a standard dose of heparin-warfarin. The TEE angle returned to its previous angle. The TEE showed dark AAo thrombi (Figure 6 and Video 6; 8.5 months had passed from the start). The serum D-dimer concentration was 0.9 μg/ml (normal: < 1.0 μg/ml), and his HbA1c was 6.8%.

**Video 6:** TEE images showing AAo thrombi and approaching several white parts TEE revealed no thrombi in the AAo. There were white thrombi around the ostia of the RMPV, which seemed to approach the wall of the AAo via line-like white thrombi, which did not move with heartbeats. Some areas of the line-like white thrombi showed long white shadows, suggesting that the white areas might contain large calcifications. The red areas in the SVC, located on the right side of the AAo, indicate blood flow in the SVC. The red areas in the LA appeared to have no obstruction. Red blood flow from the RMPV and RUPV moves straight up, indicating the absence of thrombi in the LA. TEE: transesophageal echocardiography, AAo: ascending aorta, LA: left atrium, RLPV: right lower pulmonary vein, RUPV: right upper pulmonary vein, SVC: superior vena cava

During these treatments, no AIS or AMI was observed. Cardiac CT could not identify LA thrombi or AAo thrombi. The treatment time course is summarized in Table 1.

**Table 1:** Time course of treatment This table shows the time course of heparin-warfarin treatment and the changes in thrombus size, HbA1c, BNP, and D-dimer levels. AAo: ascending aorta, LA: left atrium, DM: diabetes mellitus, HbA1c: glycated hemoglobin, BNP: brain natriuretic protein

Duration	Total duration	Thrombus treatment	AAo thrombi	LA thrombi	D-dimer	DM medication	HbA1c	BNP
Start	0 weeks	Clopidogrel, cilostazol	4 cm × 2 cm	5 cm × 4 cm	1	Vildagliptin, metformin, dapagliflozin	6.6	10.1
3 weeks	3 weeks	Clopidogrel, cilostazol, heparin	3 cm × 1.5 cm	5 cm × 3.5 cm	1	Dapagliflozin	6.8	No test
3 weeks	6 weeks	Warfarin, heparin	1.5 cm × 1 cm	4.5 cm × 3.5 cm	No test	None	No test	No test
4 weeks	2.5 months	Warfarin, heparin	1 cm × 0.5 cm	5 cm × 3 cm	1	None	7.2	No test
5 months	7.5 months	Warfarin	No detectable	Not seen	No test	None	7	35.2
4 weeks	8.5 months	Warfarin, heparin	Resolved	(Mostly) resolved	0.9	None	6.8	37.2

## Discussion

Three weeks of initial heparin-warfarin treatment improved the patient's T2DM, reducing DM medications from 3 to 1. I report here, for the first time, that more than three weeks of additional heparin-warfarin treatment further decreased the patient's DM medication use to 0. Traditional complications of DM include macrovascular diseases, such as AMI and AIS, and microvascular diseases, including limb ischemia, nephropathy, retinopathy, and peripheral neuropathy. To prevent these complications, we need to minimize blood glucose levels as much as possible without risking hypoglycemia. In the present case, hyperglycemia and hypoglycemia did not occur after stopping DM medications; therefore, heparin-warfarin therapy could be the best treatment for mild to moderate T2DM.

In my previous paper, I reported that RUPV thrombi and AAo thrombi were connected with line-like thrombi located under the SVC [11]. The line-like white thrombi approach the AAo wall and may affect it. In the present case, similar AAo wall changes were suspected. Video 6 clearly shows that these connections started from right middle pulmonary vein (RMPV) thrombi, which had white line-like shapes between RMPV and AAo thrombi, and several parts of the line-like white thrombi had wide (5-6 mm), long (> 4 cm) white shadows, indicating large calcifications. In my previous article, I described similar line-like white thrombi between RUPV thrombi and AAo thrombi [11]. These similar line-like white thrombi might have been formed along the direction of the genome via leukocytes, as their positions and shapes were very similar; therefore, it is unlikely that these thrombi formed accidentally. In Video 1, narrow (1 mm) and short (1 cm) white shadows were sometimes identified in the RLPV, indicating powder-like calcifications. AAo thrombi had no areas with shadows.

Two types of thrombus calcification, namely, large and powder-like calcifications, have been reported in the literature. Large calcified thrombi contain many CD45+/CD68+/α-SMA+ cells [12], which have been reported to transdifferentiate into myofibroblasts [13]. These CD45+/CD68+/α-SMA+ cells could differentiate into smooth muscle cells [14], potentially affecting the smooth muscle of the AAo wall.

Surrounding the entrances of the RUPV and RMPV are many white areas with strong white shadows (Video 1, Video 2, and Video 4), indicating that these areas may contain large calcified thrombi with many CD45+/CD68+/α-SMA+ cells, which are thought to be myofibroblasts. White thrombi around the ostia of the RUPV appeared to connect to AAo thrombi through line-like white thrombi. These CD45+/CD68+/α-SMA+ cells could differentiate into smooth muscle cells [14] and might interfere with the smooth muscle of the AAo wall via line-like white thrombi; therefore, line-like white thrombi might attach to the AAo wall. Recently, a hemangioma over the right coronary artery was reported around a line-like white thrombus position [15]. This hemangioma might be derived from myofibroblastoma, as evidenced by line-like white thrombi. PVTs might directly affect surrounding tissues via leukocytes in thrombi. Several tumors might be associated with third-type thrombi (line-like white thrombi that form outside vessels) and were first described. These tumors could be inhibited by resolving line-like white thrombi using warfarin or DOACs. To clarify these mechanisms, more studies are needed.

Additionally, other groups have shown that retrieved thrombi contain CD34-positive cells [16]. CD34-positive cells can transform many cell types, including smooth muscle cells and endothelial cells. CD34-positive cells may also interfere with AAo wall changes. The appearance of the AAo wall changed at the attached areas, where line-like white thrombi approached (Figure 1 and Video 1). In 2013, Nishizaki et al. reported that AAo thrombi caused AMI [17], which were near the position of my reported AAo thrombi. Their AAo thrombi could be detected on enhanced CT because they lacked areas of enhancement, unlike the AAo thrombi in the present case. Interestingly, the endothelial cells near the thrombus attachment area were CD34-positive. These CD34-positive endothelial cells may originate from CD34-positive cells in the thrombi, which connect to RUPV thrombi. These possible relationships are interesting and crucial because of the new role of PVTs and the inclusion of CD34-positive cells, which are uncertain; therefore, more studies are needed to confirm these relationships.

Extended thrombi from PVTs may affect the AAo wall, potentially leading to AAo atherosclerosis; we also need to determine their effects on other organs, such as the coronary arteries. I reported that left atrial diverticula (LADs) were present near the attachment area of extended LA thrombi from the RLPV. The origin of LADs is unclear. Myofibroblastoma and/or CD34-positive cells may differentiate into LA wall cells and form LADs. I also report atypical vessels arising from the proximal right coronary artery (RCA: #1) and the LADs [18]. Atypical vessels may form from cells within thrombi, such as myofibroblasts and CD34-positive cells. Many more studies are needed to clarify these issues.

The patient had elevated homocysteine levels, which are associated with thrombus formation and an increased risk of AMI, AIS, and deep thromboembolism [19]. The elevated homocysteine levels might have affected the results of the present patient. For example, hypoglycemia occurred two weeks after the start of treatment; however, hypoglycemia usually occurred after one week, indicating that homocysteine could block the effect of heparin treatment. Heparin-warfarin, with continued warfarin use, can ameliorate mild to moderate T2DM for a long time, indicating that mild to moderate T2DM is caused by microthrombi. Gestational DM patients with hypertension or antiphospholipid syndrome (APS) are treated with low-molecular-weight heparin, and NETs are associated with gestational DM [20]. Heparin seemed to ameliorate insulin efficiency by destroying NETs; therefore, heparin-warfarin treatment caused hypoglycemia one or two weeks after the start of heparin-warfarin treatment, and we needed to decrease the number of DM medications. In the present case, hypoglycemia occurred within two weeks. The reasons for these differences in duration might include variations in the number or structure of NETs and in NET-associated microclots, which high homocysteine concentrations might cause. More studies are needed to clarify these relationships. Nevertheless, additional treatment with heparin-warfarin could also improve insulin efficacy.

Limitations

This is only one case report; therefore, it has limited generalizability. It is challenging to measure LA and AAo thrombi accurately because their shapes are irregular; therefore, the measurement results should be approximate. Changes in the AAo wall were observed only using TEE; these changes should be examined with other modalities, including microscopy, and genome studies are needed. Both the long-term effects of warfarin and those of heparin-warfarin treatment on patients with severe T2DM are unclear, and further research is needed to address these gaps.

## Conclusions

Additional treatment with heparin-warfarin could improve insulin efficacy. A standard heparin-warfarin dose could be a promising treatment for T2DM, maintaining normal blood glucose levels without hypoglycemia. Treatment partially resolved AAo thrombi, which were connected to RMPV thrombi through line-like white thrombi near the SVC. The line-like thrombi appeared to approach the AAo wall and affect its structure.
